# The Novel Protein Cj0371 Inhibits Chemotaxis of *Campylobacter jejuni*

**DOI:** 10.3389/fmicb.2018.01904

**Published:** 2018-08-15

**Authors:** Xueqing Du, Ke Kong, Hong Tang, Haiyan Tang, Xinan Jiao, Jinlin Huang

**Affiliations:** Jiangsu Key Lab of Zoonosis, Jiangsu Co-Innovation Center for Prevention and Control of Important Animal Infectious Diseases and Zoonoses, College of Bioscience and Biotechnology, Yangzhou University, Yangzhou, China

**Keywords:** *Campylobacter jejuni*, Cj0371 protein, protein interaction, chemotaxis protein, chemotaxis pathway

## Abstract

*cj0371* is a novel gene that is associated with *Campylobacter jejuni* virulence, and an isogenic mutant of *cj0371* showed hyper chemotaxis and motility. Chemotactic motility is an important virulence factor and is involved in *C. jejuni* pathogenesis. *Campylobacter* sp. has specific variations of the common chemotaxis components, including histidine autokinase CheA, coupling scaffold protein CheV, chemotaxis response regulator protein CheY and several chemoreceptor proteins. In this study, we used immunoprecipitation combined with LC-MS/MS analyses to screen six chemotaxis pathway proteins that potentially interact with the putative protein Cj0371. qRT-PCR was used to quantitatively analyze the expression of these chemotaxis genes and basic flagella genes. The results showed that the expression of *cheV*, *cj1110c*, and *cj0262c* was significantly up-regulated, and four flagella genes also had up-regulated expression in the *cj0371* mutant. GST pull-down analyses found that Cj0371 interacted with the receiver domain of the CheV protein. Enzyme-coupled spectrophotometric assays showed that the ATPase activity of CheA was higher when Cj0371 was not present in the chemotaxis reaction medium. Therefore, we concludes that *cj0371* has a negative influence on *C. jejuni* chemotaxis, which may occur by adjusting the receiver domain of CheV to influence chemotaxis. This paper provides a new component in the chemotaxis pathway of *C. jejuni* for the first time and highlight the complexity of this remarkable pathway.

## Introduction

*Campylobacter jejuni* is the causative agent of acute bacterial gastroenteritis in humans (Hoang et al., 2011). In the past decade, human infections with the zoonotic pathogen have progressively risen in developed as well as developing countries (Bereswill et al., 2016; Berradre-Saenz et al., 2017). According to previous data, more than 80% of confirmed cases of campylobacteriosis were reported to be associated to *Campylobacter jejuni* (Helwigh et al., 2015). The clinical manifestation of campylobacteriosis is severe gastroenteritis (Bronnec et al., 2016). Infected individuals may be asymptomatic or exhibit rather mild symptoms like watery diarrhea, whereas other patients suffer from acute ulcerative enterocolitis with inflammatory bloody diarrhea. On rare occasions, reactive arthritis or neurological complications including Guillain–Barré and Miller–Fisher syndromes may arise with a latency of several weeks to several months postinfection (Wakerley et al., 2014).

The recognized pathogenic mechanisms of *C. jejuni*, including motility, chemotaxis, host cell adhesion and invasion, as well as the production of toxin, are mediated by several virulence factors (Flanagan et al., 2009). Chemotactic motility is an important virulence factor, it is the ability of bacterial cells to detect temporal changes in the chemical concentration of their surrounding environment. Flagella-mediated motility have been reported to play an important role in the intestinal colonization of avian and the invasion of intestinal epithelial cells (Nachamkin et al., 1993; Szymanski et al., 1995). *Campylobacter* sp. has specific variations of the common chemotaxis components, including histidine autokinase CheA, coupling scaffold protein CheV, chemotaxis response regulator protein CheY and several chemoreceptor proteins such as Cj0019c, Cj1564, Cj0262c, and Cj1110c. In our previous research, we identified a novel gene *cj0371* and found it influences the invasion and colonization of *C. jejuni*, so we discerned that *cj0371* is a virulence-associated gene. In addition, we also confirmed that *cj0371* plays a negative role in motility and chemotaxis. We hypothesize that *cj0371* is a new virulence-associated gene and, by influencing chemotaxis, plays a negative role in *C. jejuni* pathogenicity (Du et al., 2016).

In this paper, we continue to explore the function of Cj0371 protein. First, we carried out co-immunoprecipitation (co-IP) coupled to LC-MS/MS analysis to discover the proteins that interact with Cj0371. Then, we selected chemotaxis pathway proteins as one of the emphases for research, and used qRT-PCR to quantify the expression level of these possible interactive chemotaxis proteins. We used GST pull-downs to validate predicted protein-protein interactions. Finally, we used enzyme-coupled spectrophotometric assays to detect the ATPase activity of CheA when Cj0371 protein was added to the chemotaxis reaction medium. Our study presents an insight into the pathogenic mechanism of *C. jejuni* and offers exciting new directions for future research.

## Materials and Methods

### Bacterial Strains, Media, and Culture Conditions

The complete list of bacterial strains and plasmids used in this study are described in **Table 1**. *C. jejuni* Δ*cj0371* and *C. jejuni Δcj0371*+ strains were described in our previous report (Du et al., 2016). *E. coli* DE3 expressing GST-Cj0371 and 6 × His-Cj0371 protein were preserved in our laboratory. *C. jejuni* strains were routinely grown on Campy blood-free selective medium (CCDA; Oxid Ltd., United Kingdom) or Mueller-Hinton agar (MH; BD Ltd., United States) microaerobically [85% N_2_ (v/v), 10% CO2 (v/v), and 5% O_2_ (v/v)] in a jar at 42°C. *E. coli* DH5α and DE3 (BL21) that were used for cloning and expressing *C. jejuni* proteins were routinely cultured in Luria-Bertani (LB) agar plates at 37°C overnight. When required, LB broth was supplemented with ampicillin (100 μg/mL), kanamycin (50 μg/mL) and chloramphenicol (30 μg/mL). The *C. jejuni* transformants were selected on plates supplemented with 50 μg/mL kanamycin and/or 20 μg/mL chloramphenicol, and all *C. jejuni* strains were stored at -70°C in brain heart infusion broth (BHI; BD Ltd., United States) containing 30% glycerol.

**Table 1:** Bacterial strains and plasmids used in this study.

Strains or plasmids	Genotype	Source or reference
**Strains**		
***E. coli***		
DH5α	*endA1 hsdR17* (r_K_^-^ m_K_^+^) *supE44 thi-1 recA1 gyrA relA1* Δ(*lacZYA-argF*)*U169 deoR* [*Φ80dlac* (*lacZ*Δ*M15*)]	Invitrogen
BL21 (DE3)	F- *ompT hsdSB* (rB- mB-) *gal dcm*	Novagen
DE3 (GST)	BL21(DE3) (pGEX-6P-I)	This study
DE3 (GST-Cj0371)	BL21(DE3) (pGEX-6P-I-*cj0371*)	This study
DE3 (His-Cj0371)	BL21(DE3) (pET-30a-*cj0371*)	This study
***C. jejuni***		
*C. jejuni* 11168	*C. jejuni* NCTC11168	This study
*C. jejuni*Δ*cj0371*	*C. jejuni* NCTC11168 *cj0371::cm^r^*	This study
*C. jejuni*Δ*cj0371*+	*C. jejuni* NCTC11168 *cj0371::cm^r^* (pRY107-P*metK*-*flag*-*cj0371*)	This study
**Plasmids**		
pET-19b	Expressing His-tagged vector, *AmpR*	Novagen

### Immunoprecipitation of Interacting Proteins and LC-MS/MS Analysis

The *C. jejuni* strain expressing a FLAG-Cj0371 protein was constructed as descried in our previous study (Du et al., 2016). The steps were identical to the complementation of the *cj0371* mutation in *C. jejuni*Δ*cj0371*. The only difference was the use of different specific primers; the flag sequence was added by the forward primer in this study. The *C. jejuni* strain expressing a FLAG-Cj0371 protein was grown on two Mueller-Hinton agar plates for approximately 24 h, resuspended in PBS, pelleted at 5,000 rpm, and then resuspended in 2 mL of lysis buffer (50 mM Tris HCl, PH 7.4, 150 mM NaCl, 1 mM EDTA, 1% Triton X-100), 1% Triton X-100, and protease inhibitors (ROCHE). After lysis by sonication, cell debris was removed by centrifugation at 12,000 rpm, supernatants were recovered, and immunoprecipitation of FLAG-Cj0371 protein was performed using anti-FLAG M2 affinity gel following the manufacturer’s recommendations and methods described in a previous report (Gao et al., 2014). In brief, the ANTI-FLAG M2 affinity gel was thoroughly suspended in the vial to achieve a uniform suspension of the resin. A 40 μL volume of the gel suspension was transferred to a 1.5 mL tube. The resin was centrifuged at 5,000 ×*g* for 30 s. The supernatant was removed and the resin was washed three times with TBS (50 mM Tris-HCl, 150 mM NaCl, pH 7.4). Then, 200 μL of cell lysate was added to the washed resin and brought to a final volume of 700 μL by the addition of lysis buffer. All samples were shaken for 3 h at 4°C. After incubation, the resin was washed six times with 0.5 mL of TBS and eluted with 100 μL of 0.1 M glycine HCl, PH 3.5. All elution products were pooled and loaded onto 12% SDS-PAGE gel, and the gel pieces was sent to Protech in Suzhou for LC-MS/MS.

### RNA Preparation and Real-Time PCR

The wild type *C. jejuni* 11168 and the previously described line *C. jejuni* Δ*cj0371* were grown in Mueller-Hinton broth (MH, BD, United States) under microaerobic conditions at 42°C with shaking (180 rpm) and harvested at the stationary (18 h) phase of growth. *C. jejuni* bacteria pellets were resuspended in RNAprotect Bacteria Reagent (Qiagen) to preserve them. For RNA preparation, bacteria pellets were resuspended in Qiagen RNeasy lysis buffer containing β-mercaptoethanol and shocked in a vortex for 30 s. The RNA was further purified using the Qiagen RNeasy Plus mini kit as described previously and in the manufacturer’s instructions (Chandrashekhar et al., 2015). The absence of DNA was confirmed by PCR amplification using *Campylobacter*-specific 16S rRNA primers, and total RNA was quantified in a NanoDrop 1000 device (Thermo Scientific, Germany). cDNA was prepared from equivalent amounts (1 μg) of total RNA using the PrimeScript^TM^ RT-reagent kit with gDNA Eraser (Takara) according to the manufacturer’s instructions.

Quantitative RT-PCR was performed using SYBR green mix (Roche) on a 2 μL volume of cDNA and normalized to the results of a corresponding 16S rRNA gene control reaction mixture prepared from the same cDNAs (primers used for RT-PCR are listed in **Supplementary Tables S1**, **S2**). Reactions were carried out in a 7500 Real Time PCR system [Applied Biosystems (ABI, United States)]. Cycling conditions were as follows: denaturation for 10 min at 95°C and amplification for 40 cycles at 95°C for 10 s, 59°C for 30 s, and 72°C for 30 s. For DNA quantification, a serial standard was prepared from a purified PCR product of the gene of interest. Each reaction was performed in triplicate. Samples were tested in triplicate, and the analysis was replicated at least three times. Relative gene expression was determined by the 2^-ΔΔC_T_^ method (Livak and Schmittgen, 2001).

### Recombinant Expression and Purification of *C. jejuni* Proteins

CheA, CheY, CheV, Cj1110c, Cj1564, Cj0262c, and Cj6462 proteins were expressed by the pET-19b plasmid in the DE3 system. We used a one-step cloning kit to generate the recombinant expressing plasmids following the manufacturer’s recommendations and methods described in a previous report. The genes *cheA*, *cheY*, *cheV*, *cj1110c*, *cj1564*, *cj0262c*, and *cj1564* were amplified by PCR from *C. jejuni* 11168 using specific primers which had homologous sequence with pET-19b, with the restriction sites *Nde* I in the forward primers and *Bam*H I in the reverse primers (**Supplementary Table S3**). Then, the fragments were cloned into pET-19b using one-step cloning kit. The recombinant plasmids were used to transform *E. coli* DH5α. Transconjugants were selected on plates containing Ampicillin (100 μg/μL). The positive transconjugants were confirmed by PCR analysis and nucleotide sequencing. The plasmids extracted from DH5α were transferred into DE3 (BL21) to express.

To determine the expression of different proteins from individual clones, preliminary expression was carried out as described in previous reports (Suryanarayana et al., 2016; Faber et al., 2016). The recombinantly expressed CheA, CheY, CheV, Cj0371, and Cj1564 (317–662 residues) proteins contained 6 × His tags on their N termini and were purified from the soluble bacterial fraction by affinity chromatography using Ni2-nitrilotriacetic acid (NTA)-Sepharose beads (Novagen) under native conditions. The purification procedure was modified according to the manufacturer’s recommendations for the His Bind Purification Kit and methods described in a previous report (Novagen, Madison, WI, United States) (Sheng et al., 2007). The eluted proteins were analyzed by SDS-PAGE, pooled and dialyzed against at least three changes of PBS using a dialysis chamber (biosharp, 50 kDa).

### Anti-GST Tag Pull Down Assay

As described in a previous report, to investigate protein interactions with Cj0371, GST pull-down analysis was performed (Vikis and Guan, 2004). A volume of 200 μL of the soluble fractions of DE3 (pGEX-6P-I-*cj0371*) or DE3 (pGEX-6P-I) was transferred to mini spin columns as the bait protein and 300 μL PBST (add 1% TritonX-100 to PBS) was added, then the columns were incubated for 3 h at 4°C with 50 μL of Anti-GST beads (Thermo Scientific). Then, the resin was washed six times with 500 μL PBST. After that, 200 μL soluble fraction of DE3 (pET-19b-*cheV*) or other recombinant strains was added to the columns and incubated for 6 h at 4°C. The resin was washed with PBST again approximately 10 times. Bound proteins were eluted with 60 μL 10 mmol glutathione. The eluted proteins were analyzed by Western blotting using the anti-His monoclonal antibody as the primary antibody.

### CheA Protein ATPase Activity Assay

We investigated the effect of Cj0371 in the chemotaxis pathway using a coupled enzyme assay, which was based on a reaction in which the regeneration of hydrolyzed ATP is coupled to the oxidation of NADH (Sehgal et al., 2016). CheA ATPase activity was assayed using an enzyme-coupled spectrophotometric assay as described by previous reports with some modifications (Ninfa et al., 1988, 1991). The reaction was performed in 100 μL of a basic medium containing: 100 mM K_3_PO_4_, PH 7.5, 5 mM Mgcl_2_, 0.1 mM EDTA, 0.1 mM DTT, 10 mM ATP, 0.2 mM NADH, 1 mM PEP, 2 U PK (Sigma), and 6.6 U L-LDH (Roche). The chemotaxis proteins mix volume was 100 μL, if one protein was not put in the mix, and brought to a final volume with deionized water. At the beginning of the reaction, the reaction mix was equilibrated at ambient temperature. The reaction was conducted at ambient temperature, and NADH oxidation was monitored in a Microplate Reader recording the absorbance at 340 nm every 30 s. According to a previous report, we selected the chemotaxis proteins mix including 0.2 μM CheA, 10 μM CheY, 4 μM CheV, and 1 μM Cj1110c (Levit et al., 1998). The reactions were initiated by the addition of chemotaxis proteins mix, and the course of the reaction was monitored until a stable linear decay of absorbance was observed. The rate of decay over 15–30 min was used to indicate ATP hydrolysis rates. The control experiment was the reaction in the absence of any protein.

### Statistical Analysis

Data analysis was performed using GraphPad. Statistical significance of the data was determined using Student′s *t*-test in cases, where only two data sets were compared. The values of *P* < 0.05 was considered statistically significant.

## Results

### Cj0371 May Play a Role in Chemotaxis Pathway of *C. jejuni*

Co-immunoprecipitation followed by liquid chromatography-mass spectrometry (LC-MS/MS) analysis is a useful way to screen interacting proteins (Schieltz et al., 2006). We selected this strategy to screen for proteins that interact with Cj0371. The screened proteins were classified according to the pathway (KEGG Pathway Database). We screened approximately 18 proteins, 2 proteins in the two-component system, 6 proteins in the bacterial chemotaxis pathway (which we call chemotaxis proteins in this paper), 5 proteins in metabolic pathways (including glucose metabolism, nucleic acid metabolism, and protein metabolism), 2 proteins in RNA degradation, 2 proteins in the bacterial secretion system, and 1 flagella protein, FlgE (**Table 2**). However, not all the proteins identified by screening actually interact with Cj0371. A few proteins in same pathway were identified, that may have a cascade of interactions between proteins, and so we reasoned that Cj0371 may play a role in these pathways. According to our previous research, we found when *cj0371* was inactivated, chemotaxis and motility of *C. jejuni* was increased. Therefore, in this paper we focused on the chemotaxis pathway of *C. jejuni* for further research.

**Table 2:** Proteins that probably interact with the Cj0371 proteins identified in this study.

Pathway	NCTC1168 gene	Annotation	Proteins mass (Da)	No. of peptide
Two-component system	*cj1487c*	ATP Binding	31162	1
	*cj0694*	Isomerase activity	57507	1
Bacterial secretion system	*cj0942c* (*secA*)	Protein transport, translocation	97976	3
	*cj0958c* (*yidc*)	Membrane protein insertase	60828	1
ABC transporters	*cj0773c*	Amino acid-transporting ATPase activity	33504	1
	*cj0175c*	Periplasmic iron-binding protein	37476	1
Flagellar assembly	*cj0043* (*flgE*)	Bacterial-type flagellum basal body	58231	1
Metabolic pathways	*cj0835c* (*acnB*)	Aconitate hydratase activity	92793	1
	*cj1437c*	Sugar transferase	41929	1
	*cj1487c*	Oxidative phosphorylation	31162	1
	*cj1037c*	ATP binding pyruvate carboxylase activity	54413	2
	*cj0933c*	Ribose-phosphate pyrophosphokinase	65834	8
Bacterial chemotaxis	*cj0285c* (*cheV*)	Chemotaxis protein	35858	9
	*cj0284c* (*cheA*)	Chemotaxis protein	85300	3
	*cj0019c*	Methyl-accepting chemotaxis protein	66845	1
	*cj1564*	Methyl-accepting chemotaxis protein	73121	2
	*cj0262c*	Methyl-accepting chemotaxis protein	72831	1
	*cj1110c*	Methyl-accepting chemotaxis protein	48351	2

### RT-qPCR Quantification of Chemotaxis and Flagella Genes

**FIGURE 1:**
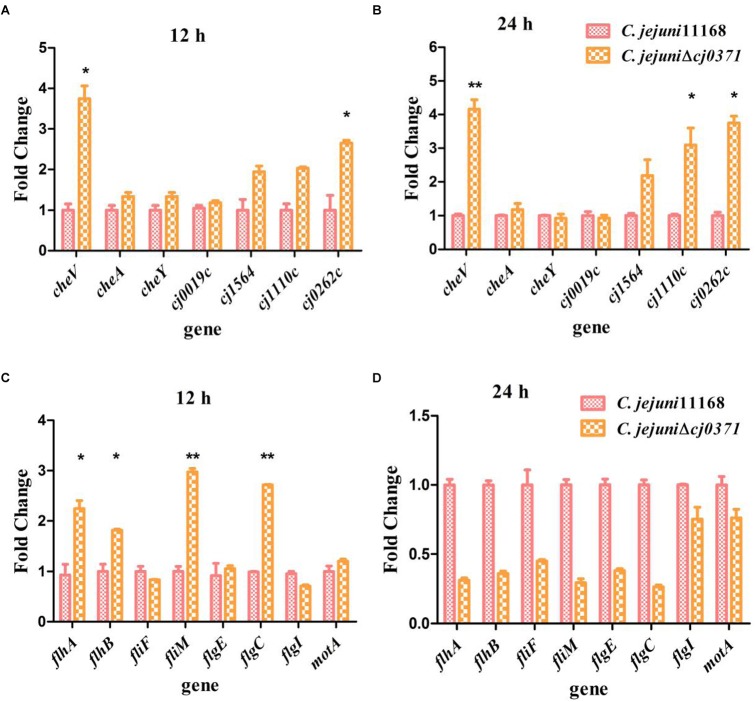
Analysis of the relative expression of chemotaxis genes and basal flagellar genes by real-time PCR. The relative expression of major chemotaxis pathway genes (including these chemotaxis genes that screened by co-IP) at 12 h of *C. jejuni* growth **(A)** and stationary phase (24 h) **(B)**. **(C,D)** Show the relative expression of flagellar base genes at 12 h and 24 h of *C. jejuni* growth. *C. jejuni* RNA was reverse-transcribed to cDNA, and the expression values of all chemotaxis genes and flagellar genes are represented as relative to wild-type levels and normalized to 16S RNA. Mean values and standard deviations from triplicate measurements are shown. Significant *P-*values are indicated by asterisks (Student’s *t-*test, unpaired, one-sided) as follows: ^∗^0.01 < *P* < 0.05; ^∗∗^0.001 < *P* < 0.01.

RT-qPCR was used to confirm the differential expression of major chemotaxis pathway genes (including these chemotaxis genes that screened by co-IP) and the flagellar pathway genes that are regulated by chemotaxis. Whether in log (at 12 h of *C. jejuni* growth) or stationary phase (24 h), *cheV*, *cj1564*, *cj0262c*, and *cj1110c* all showed increased expression in *C. jejuni* Δ*cj0371*. Especially, compared to the wild type *C. jejuni* 11168, *cheV* had significantly increased expression in *C. jejuni* Δ*cj0371* (12 h, *P* < 0.05; 24 h, *P* < 0.01; **Figures 1A,B**). The flagellar motility of *C. jejuni* is controlled by chemotaxis. The transition between counterclockwise and clockwise rotation is controlled by proteins located at the base of the flagellar motor (Welch et al., 1993). Therefore, we also characterized flagellar base genes differential expression by RT-qPCR. These flagellar genes included the genes encoding flagellar hook (*flgE*), C ring (*fliM*), MS ring (*fliF*), P ring (*flgI*, *flgC*), flagellar output system (*flhA*, *flhB*) and flagellar motor protein (*motA*). In the log phase of *C. jejuni* growth, the flagellar genes *flhA*, *flhB*, *fliM*, and *flgC* showed increased expression in the *C. jejuni* Δ*cj0371* (**Figures 1C,D**). In stationary phase, it may be that *cj0371* mutants enter the declining period earlier than the wild type strain.

### Interaction of Cj0371 Proteins With Screened Chemotaxis Proteins

**FIGURE 2:**
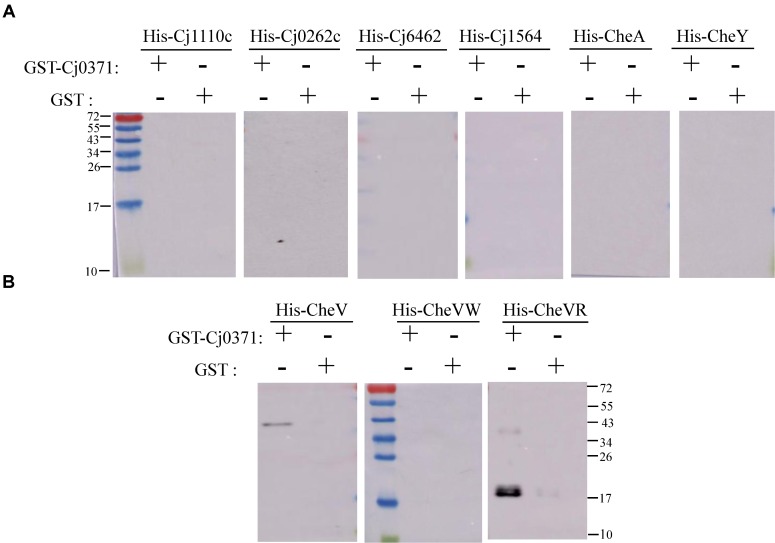
Confirmation of interactions between chemotaxis proteins and Cj0371 by Western blot analysis. No interaction was detected between Cj0371 and CheA, CheY, Cj1110c and other polypeptides **(A)**. The interaction between Cj0371 and CheV, the regulatory domain and the CheW-like domain of CheV **(B)**. The size of the pre-stain marker is marked in numbers, and the unit is kDa. His-tagged chemotaxis proteins were co-eluted with GST-tagged Cj0371 when assays, described in the experimental procedures, were performed with cell lysates overexpressing both fusion proteins. His-tagged proteins expressed by DE3 (pET-19b) were analyzed by Western blotting using an anti-His antibody.

To provide additional evidence for a potential direct role of Cj0371 in chemotaxis, we searched for the interaction of chemotaxis proteins identified in our screen with Cj0371 protein. First, we used the DE3 (pET-19b) expression system to express 7 proteins or polypeptides including CheA, CheV, CheY, Cj1110c, Cj1564 (residues 42–290 of Cj1564), Cj0262c (residues 1–319 of Cj0262c) and Cj6462 (This paper named. It is the residues 317–662 of Cj1564, which are identical to residues 320–665 of Cj0262c.). The expression of proteins or polypeptides were analyzed by SDS-PAGE and Western blotting with an anti-His monoclonal antibody (**Supplementary Figure S1**). Then, we detected the interaction between Cj0371 and these chemotaxis proteins by GST pull-down analysis. The results showed that Cj0371 only interacts with CheV. No interaction was detected with CheA, CheY, Cj1110c, and other polypeptides (**Figures 2A,B**). Other screened chemotaxis proteins were identified to be false positive proteins from the co-IP analysis. When the washing strength of co-IP analysis was weak, many cascade proteins were eluted with first protein that interacted with Cj0371. The screened false positive proteins are not useless because by assessing the proteins they are in the same pathway, we can infer the possible pathways in which unknown proteins may play a role.

CheV has two domains: the CheW-like domain and the receiver (REC) domain. The CheW-like domain is crucial for physically connecting chemoreceptors to the CheA kinase, and phosphorylation of the CheV receiver domain might adjust the efficiency of its coupling and thus allow the system to modulate the response to chemical stimuli in an adaptation process (Alexander et al., 2010). We also used the DE3 (pET-19b) expressing system to express the two domains of CheV, and used GST pull-down to detect which domain interacts with Cj0371. The results showed that Cj0371 interacts with the regulatory domain of CheV rather than the CheW-like domain (**Figure 2B**). Therefore we inferred that Cj0371 may regulate the receiver (REC) domain of CheV to influence chemotaxis.

### Cj0371 Inhibits the ATPase Activity of CheA

**FIGURE 3:**
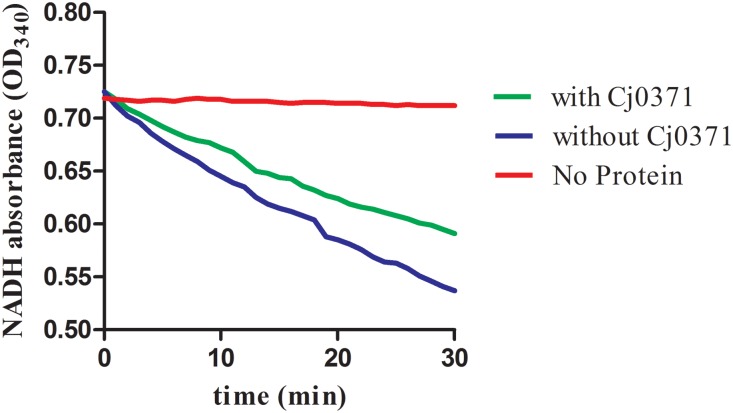
The ATPase activity of CheA protein measurements by an enzyme-coupled spectrophotometric assay. “No protein” indicates the reaction mix with no chemotaxis proteins added (red line). With or without Cj0371 indicates when CheA, CheV, and Cj6462 (317–662 residues of Cj1564) were all present in the chemotaxis reaction medium, with or without Cj0371 protein in the medium.

CheA is a chemotaxis histidine kinase. It contains the known CheA functional domains: a phosphorylation site (P1 domain), a catalytic kinase domain, a receptor-docking region (Bilwes et al., 1999). We purified chemotaxis proteins and Cj0371 (**Supplementary Figure S2**), then simulated the chemotaxis system of *C. jejuni in vitro* and detected the ATPase activity of CheA with and without Cj0371 protein in the chemotaxis reaction medium. The results showed that the reaction curve is broadly horizontal when any protein was in the basic medium (red line; **Figure 3**), this is an important negative control to ensure that changes in the mix are caused by chemotaxis proteins. The expected decreases were rapidly generated when the chemotaxis protein mixes were added to the reaction. In the absence of Cj0371, the rate of NADH decrease is faster than when Cj0371 was added to the reaction, which demonstrates that Cj0371 influences the ATPase activity of CheA and that the influence is negative.

## Discussion

**FIGURE 4:**
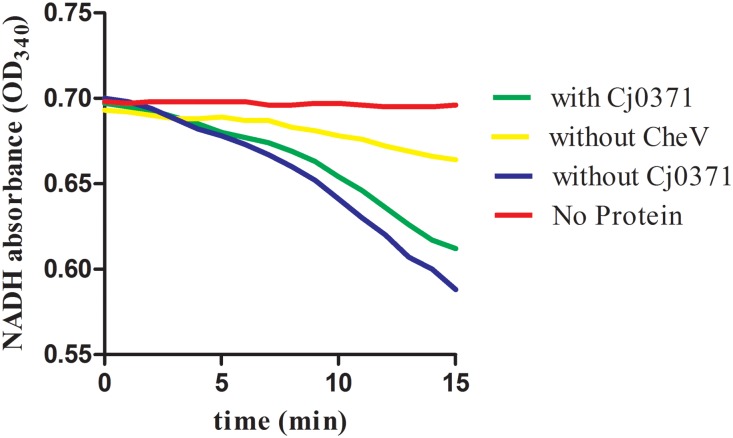
The ATPase activity of CheA protein measured by an enzyme-coupled spectrophotometric assay. With or without CheV indicates that CheA, CheV, Cj6462, and Cj0371 were all in the chemotaxis reaction, with or without CheV protein in the medium.

*C. jejuni* Δ*cj0371* displays hypermotility, enhanced growth kinetics, and increased invasion and colonization ability. It is interesting to note that *cj0371* influences *C. jejuni* chemotaxis. Bacteria motility is not only regulated by flagella but also controlled by chemotactic factors. Bacteria use a signaling cascade of protein phosphorylation and dephosphorylation reactions to control bacterial motors in response to environmental chemical changes (Miller et al., 2009). Therefore, we speculated the increased chemotaxis may result from other changes in the *cj0371* mutant. Based on of previous research, this study continued to investigate the function of the *cj0371* gene. Due to weak washing intensity in the co-IP experiments, approximately 20 putative proteins were screened, but not all of the screened proteins interact with Cj0371. Interestingly, there were six chemotaxis proteins in these screened proteins, including CheA, an important histidine kinase that receives and conducts signals in the chemotaxis pathway, CheV a scaffold protein that assists in signal conduction, as well as Cj1564, Cj0262c, Cj0019c, and Cj1110c, which are all MCP proteins that sense environmental signal. In the chemotaxis pathway, these six chemotaxis proteins are all important component. Using the *cheV* isogenic mutants in the nutrient-depleted chemotaxis assay, it showed significantly reduced chemotaxis compared with wild-type cells toward L-serine (*P* < 0.05), and the *cheV* isogenic mutants also (9.3 mm ± 2.2; *P* < 0.05) displayed significantly reduced motility compared with *C. jejun*i 11168-O wild-type (19.2 mm ± 2.2) when examined in a 0.5% Brucella agar motility assay (Hartley-Tassell et al., 2010). In our experiments testing the ATPase activity of CheA protein measurements with an enzyme-coupled spectrophotometric assay, we found that when CheV protein was not present in the chemotaxis reaction, the rate of ATP hydrolysis is the slowest (yellow line; **Figure 4**). *cj1110c* functions through controlling energy taxis to keep signaling balanced in *C. jejuni*. Inactivation of the *cj1110c* gene, a cytoplasmic sensor with two PAS-domains, resulted in increased taxis (Reuter and van Vliet, 2013). An isogenic mutant of *cj1564* was shown to have altered phenotypic characteristics, including reduced chemotactic motility and reduced ability to adhere and invade a cultured epithelial cell line (Rahman et al., 2014). For the gene *cj0262c*, we did not find any report to explain the phenotypic characteristics of the mutant. These genes all had up-regulated expression due to the mutation of *cj0371*. Therefore, we speculated that *cj0371* hinders chemotaxis of *C. jejuni*. The mechanism of chemotaxis control of bacteria flagella is highly complex, but it can be sure that chemotaxis directly adjusts the base of flagellar proteins such as FliM. Then, this study selected seven flagellar basic proteins of *C. jejuni* for analysis. In log phase growth of *C. jejuni, fliM*, *flhA*, *flhB*, and other genes, all showed up-regulated expression in *C. jejuni* Δ*cj0371*. The results of this analysis proved that the *cj0371* gene influences the flagellar pathway of *C. jejuni*.

**FIGURE 5:**
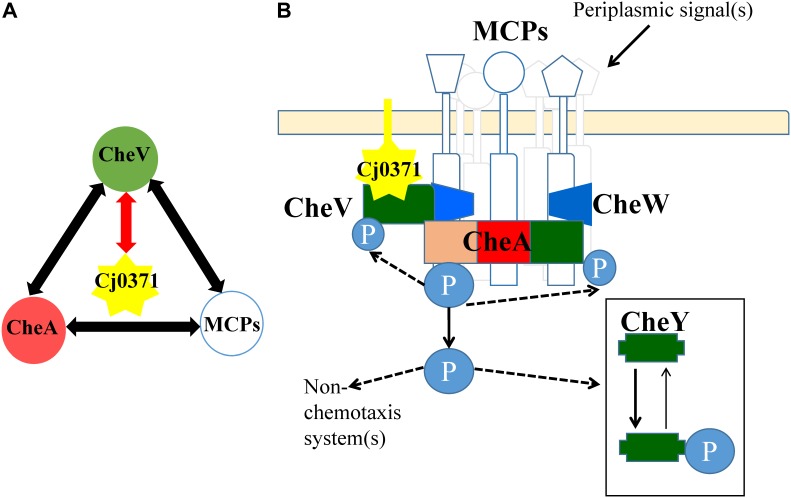
Interaction map between Cj0371 and *C. jejuni* chemotaxis proteins and a model of chemotaxis signal transduction pathways in *C. jejuni*. **(A)** Red lines with arrowheads indicate interactions confirmed by co-immunoprecipitation and GST pull-down. Other interactions are according to previous reports. **(B)** A model displaying possible signal transduction by the Cj0371 and chemotaxis proteins system, adapted from previous report (Marchant et al., 2002), and its interaction with the chemotaxis pathway via the CheV protein (see “Discussion” for more information).

In this research, only CheV interacts with Cj0371, other chemotaxis proteins were the false positive interactors from co-IP analysis. As previously reported, CheV interacts with residues 517–662 of Cj1564 (Tlp3), and this region is identical to residues 513–659 of Cj0144 (Tlp2) and 520–665 of Cj0262c (Tlp4) (Rahman et al., 2014). The interaction between CheV and CheA was previously identified by a yeast three hybrid-analysis (Hartley-Tassell et al., 2010). In addition, the phosphorylation signal transduction cascade associated with the methyl-accepting chemotaxis protein (MCP) sensor-CheA-CheW-CheY-flagellar motor backbone is a common basic principle in the chemotaxis systems of *C. jejuni* (Gegner et al., 1992; Marchant et al., 2002). Therefore, the *C. jejun*i chemotaxis pathway is a complex protein network, and the proteins interact with each other (**Figure 5A**). Taking the interaction between CheV and Cj0371 as the core interaction, it is not surprising that we screened other chemotaxis cascade-interactive proteins. Now, we can confirm that *cj0371* plays a role in the chemotaxis pathway. However, how *cj0371* works in the chemotaxis pathway and what function it plays still need more research to confirm.

To thoroughly research the function of Cj0371 protein, we simulated the chemotaxis pathway of *C. jejuni in vitro* and assayed the ATPase activity of CheA with the Cj0371 protein present or absent. We found that Cj0371 inhibited CheA from hydrolyzing ATP in the reaction medium. *cj0371* plays a negative role in the chemotaxis pathway. This result is consistent with our previous research showing that *C. jejuni* Δ*cj0371* displays hyperchemotaxis, motility and so on.

Cj0371 interacts with CheV, which is a central component of the *C. jejuni* chemotaxis signal transduction pathway. CheV consists of a CheW domain fused to a receiver domain that is capable of being phosphorylated. Further study showed that Cj0371 interacted with the receiver (REC) domain of CheV. The function of the CheV protein is not completely understood in *C. jejuni* (Zautner et al., 2012). However, we have confirmed that CheV plays a crucial role in the chemotaxis pathway. As the enzyme-coupled spectrophotometric assay showed, when CheV was not present in the chemotaxis reaction medium, the rate of ATP hydrolysis was almost zero (**Figure 4**). As the TMHMM^1^ database predicts, and consistent with the localization of cell cytoplasm and membrane analysis, Cj0371 is a transmembrane protein (**Supplementary Figure S3**). Phosphorylation of the CheV receiver domain might allow the chemotaxis pathway system to modulate the response to chemical stimuli in an adaptation process. We have shown that Cj0371 plays a role in the chemotaxis pathway. According our research and previous report model, we modified the model of *C. jejuni* chemotaxis pathway (**Figure 5B**). Whether Cj0371 protein through preventing CheV phosphorylation to decrease chemotaxis? Asp residue is important for the phosphorylation of regulators containing REC domain. In future studies, We will research the change of phosphorylation of 242 Asp residue (4-aspartylphosphate) when *cj0371* gene was inactivated.

## Conclusion

In conclusion, *cj0371* plays a negative role in the chemotaxis of *C. jejuni*. It is not good for bacterial evolution to have one gene that is able to reduce virulence or inhibit the growth and metabolism of bacteria. Therefore, why is *cj0371* stable and conserved in the genome of *C. jejuni*? In fact, some genes are called ‘anti-virulence’ genes, and multiple hypotheses have been offered to explain why these genes are retained in the genome of bacteria (Foreman-Wykert and Miller, 2003; Rasko and Sperandio, 2010). Regarding the *cj0371* gene, we prefer the view that it allows *C. jejuni* to evolve toward benign coexistence with its host, as occurs in poultry (it is well-known that poultry is an important reservoir of *C. jejuni*). Until now, there has been no explanation as to why *C. jejuni* can coexist with poultry. The function of this ‘anti-virulence’ gene may be an important reason.

Researching the function of new gene in the chemotaxis pathway, as described in this study, will contribute to understanding chemotaxis signaling pathways which are involved in colonization and its involvement in the chemotaxis pathway and its importance in the survival of this organism. This study adds a new component in the chemotaxis pathway of *C. jejuni* for the first time and provide the complexity framework for the full elucidation of the complex chemotaxis system of *C. jeju*ni. The findings in this study also provide insight into the complexity of chemotaxis receptor protein-ligand interactions with implications not just for C. jejuni chemotaxis but for all bacterial chemotaxis.

## Author Contributions

XD, JH, and XJ conceived and designed the experiments. XD, KK, HT, and HYT performed the experiments. XD analyzed the data. XD, JH, and XJ contributed reagents, materials, and analysis tools. XD wrote the paper.

## Conflict of Interest Statement

The authors declare that the research was conducted in the absence of any commercial or financial relationships that could be construed as a potential conflict of interest.

## Footnotes

1 www.cbs.dtu.dk/services/TMHMM
