# Supplementary material for: The Novel Protein Cj0371 Inhibits Chemotaxis of Campylobacter jejuni
Source: Front Microbiol. 2018 Aug 15;9:1904. doi: 10.3389/fmicb.2018.01904 (PMC6104132; doi:10.3389/fmicb.2018.01904)
Supplement: Supplementary file 1 [file Table_1.DOCX]

**TABLE S1 | The primers for the chemotaxis pathway used for qRT-PCR.**

| Genes | Primers | | Primer sequences (5'-3') | Note |
| --- | --- | --- | --- | --- |
| *cheV* | | *cheV-*F | TTCGTGGAGTGGTTATAC | Two-domain chemotaxis  coupling protein |
|  |  | *cheV*-R | AAGTTGCTGGTTCTATATCT |  |
| *cheA* | | *cheA-*F | GCTTCAGGTAGTAATGCTA | Chemotaxis kinase |
|  |  | *cheA-*R | TAACGCTCTTCAACATCA |  |
| *cheY* | | *cheY-*F | GCTTCAGGTAGTAATGCTA | Chemotaxis response  regulator protein |
|  |  | *cheY-*R | TAACGCTCTTCAACATCA |  |
| *cj0019c* | | *cj0019c*-F | CAATCTAACCCAAAGTGC | MCP protein |
|  |  | *cj0019c-*R | ATGTTCGCCTGCTCT |  |
| *cj1564* | | *cj1564-*F | GGTGATAACAACTTCTTCTCTTA | MCP protein |
|  |  | *cj1564-*R | CTACTACAATAATGACAACAATGG |  |
| *cj1110c* | | *cj1110c*-F | GAGGATGTTAATCAGAGTGT | MCP protein |
|  |  | *cj1110c-*R | TTAGCAACTTCAGCAGAG |  |
| *cj0262c* | | *cj0262c*- F | GGTGTTGTAGGTGGAGATT | MCP protein |
|  |  | *cj0262c*- R | GCATTGGCGATATTGATACTTA |  |
