# Supplementary material for: The Novel Protein Cj0371 Inhibits Chemotaxis of Campylobacter jejuni
Source: Front Microbiol. 2018 Aug 15;9:1904. doi: 10.3389/fmicb.2018.01904 (PMC6104132; doi:10.3389/fmicb.2018.01904)
Supplement: Supplementary file 2 [file Table_2.DOCX]

**TABLE S2 | The primers of the flagellar system used for real-time PCR.**

| Genes | Primers | Primer sequences (5'-3') | Amplification size | | Note |
| --- | --- | --- | --- | --- | --- |
| *fliM* | *fliM*-F | CAAACCGTGATATTATGATGGGTG | | 90 bp | Flagellar motor switch protein |
|  | *fliM*-R | ATACCACTTCAGCACGACCGA | |  |  |
| *fliF* | *fliF*-F | AATCGTTTTGTTGAGCCATTTATTC | | 130 bp | Flagellar MS-ring protein |
|  | *fliF*-R | CCTCTTGTGCTGCGACATCTTC | |  |  |
| *flhA* | *flhA*-F | TAAGCGAAGGGCAAAACGG | | 107 bp | Flagellar biosynthesis protein |
|  | *flhA*-R | AATACAAAATACAATCACGCCAATG | |  |  |
| *flhB* | *flhB*-F | GCAGGTGCGGATGTGGTG | | 129 bp | Flagellar biosynthesis protein |
|  | *flhB*-R | TTGTTTTATGCGAAGAGCGAGA | |  |  |
| *flgI* | *flgI*-F | ATGGAAGCGGCGATGGA | | 146 bp | Flagellar basal body P-ring protein |
|  | *flgI*-R | AAAGGCTGGAAGTTTGGCTG | |  |  |
| *flgC* | *flgC*-F | TGCTCAGCGTTTTAGAATGAATG | | 91 bp | Flagellar basal body rod protein |
|  | *flgC*-R | GCGTCTTCTATAAGGCCCACC | |  |  |
| *flgE* | *flgE*-F | CCATAACAACCCAATCAACCAAC | | 117 bp | Flagellar hook protein |
|  | *flgE*-R | CCACATCAAATTCGCCATCAC | |  |  |
| 16S rRNA | 16S rRNA-F | TCCACGCCCTAAACGATGTAC | | 110 bp | Reference gene |
|  | 16S rRNA-R | TGAGTTTTAATCTTGCGACCGTAC | |  |  |
