# Supplementary material for: The Novel Protein Cj0371 Inhibits Chemotaxis of Campylobacter jejuni
Source: Front Microbiol. 2018 Aug 15;9:1904. doi: 10.3389/fmicb.2018.01904 (PMC6104132; doi:10.3389/fmicb.2018.01904)
Supplement: Supplementary file 3 [file Table_3.docx]

| Primers | Primer sequences (5'-3') | Amplification size | | Restriction sites |
| --- | --- | --- | --- | --- |
| *cheV*-F | GACGACGACAAGCATATGTTTGATGAAAATATC | | 954 bp | *Nde* I |
| *cheV*-R | TTGTTAGCAGCCGGATCCTTACCCCTGTTCTTG | |  | *Bam*H I |
| *cheA*-F | GACGACGACAAGCATATGGAAGATATGCAAGAA | | 2310 bp | *Nde* I |
| *cheA*-R | TTGTTAGCAGCCGGATCCTTATCCTAGTTTCAAATT | |  | *Bam*H I |
| *cheY*-F | GACGACGACAAGCATATGGTGAAATTGTTAGTTGTT | | 393 bp | *Nde* I |
| *cheY*-R | TTGTTAGCAGCCGGATCCTTACTCAGCTGCACCTTC | |  | *Bam*H I |
| *cj1564*-F | ACGACGACGACGACAAGCATATGCTAAAAATAACAAA | | 747 bp | *Nde* I |
| *cj1564*-R | TTGTTAGCAGCCGGATCCTTAGTATTTTGATACGATGA | |  | *Bam*H I |
| *cj0262c*-F | GACGACGACGACAAGCATATGCAATCAATAAAT | | 957 bp | *Nde* I |
| *cj0262c*-R | TTGTTAGCAGCCGGATCCTTAACTTCTGCTGATTAA | |  | *Bam*H I |
| *cj6462*-F | GACGACGACAAGCATATGCTCTCCCCACTTGCA | | 1041 bp | *Nde* I |
| *cj6462*-R | TTGTTAGCAGCCGGATCCTTAAAACCTCTTCTTCT | |  | *Bam*H I |
| *cj1110c*-F | GACGACGACAAGCATATGTTTGGTGCTAAG | | 1290 bp | *Nde* I |
| *cj1110c*-R | TTGTTAGCAGCCGGATCCTTATGACATCGCTTTA | |  | *Bam*H I |

**TABLE S3 | Primers used in this study for expressing chemotaxis proteins.**
