# Supplementary material for: The Novel Protein Cj0371 Inhibits Chemotaxis of Campylobacter jejuni
Source: Front Microbiol. 2018 Aug 15;9:1904. doi: 10.3389/fmicb.2018.01904 (PMC6104132; doi:10.3389/fmicb.2018.01904)
Supplement: Supplementary file 4 [file Image_1.PDF]

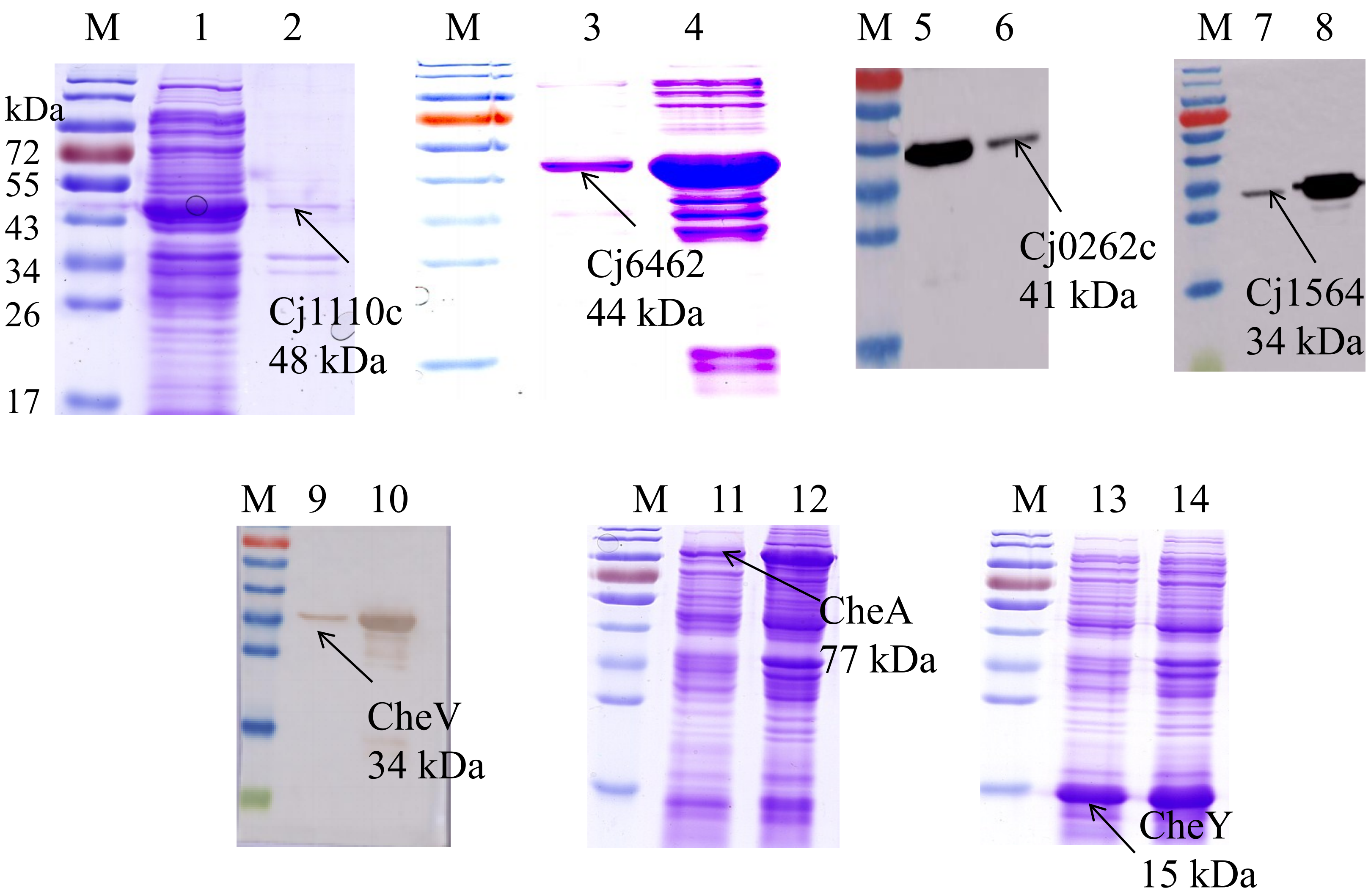
M 1 2
kDa
72
55
43
34
26
17
Cj1110c
48 kDa
M 3 4
Cj6462
44 kDa
M 5 6
Cj0262c
41 kDa
M 7 8
Cj1564
34 kDa
M 9 10
CheV
34 kDa
M 11 12
CheA
77 kDa
M 13 14
CheY
15 kDa

Fig S1 Analysis of the expression of recombinant proteins by SDS-PAGE and Western blot

M: Pre-stain maker;

1. Insoluble bacterial fraction of BL21 (DE3) (pET-19b-*cj1110c*);
2. Soluble bacterial fraction of BL21 (DE3) (pET-19b-*cj1110c*);
3. Soluble bacterial fraction of BL21 (DE3) (pET-19b-*cj6462*);
4. Insoluble bacterial fraction of BL21 (DE3) (pET-19b-*cj6462*);
5. Insoluble bacterial fraction of BL21 (DE3) (pET-19b-*cj0262c*);
6. Soluble bacterial fraction of BL21 (DE3) (pET-19b-*cj0262c*);
7. Soluble bacterial fraction of BL21 (DE3) (pET-19b-*cj1564*);
8. Insoluble bacterial fraction of BL21 (DE3) (pET-19b-*cj1564*);
9. Soluble bacterial fraction of BL21 (DE3) (pET-19b-*cheV*);
10. Insoluble bacterial fraction of BL21 (DE3) (pET-19b-*cheV*);
11. Soluble bacterial fraction of BL21 (DE3) (pET-19b-*cheA*);
12. Insoluble bacterial fraction of BL21 (DE3) (pET-19b-*cheA*);
13. Soluble bacterial fraction of BL21 (DE3) (pET-19b-*cheY*);
14. Soluble bacterial fraction of BL21 (DE3) (pET-19b-*cheY*).
