# Supplementary material for: The Novel Protein Cj0371 Inhibits Chemotaxis of Campylobacter jejuni
Source: Front Microbiol. 2018 Aug 15;9:1904. doi: 10.3389/fmicb.2018.01904 (PMC6104132; doi:10.3389/fmicb.2018.01904)
Supplement: Supplementary file 5 [file Image_2.PDF]

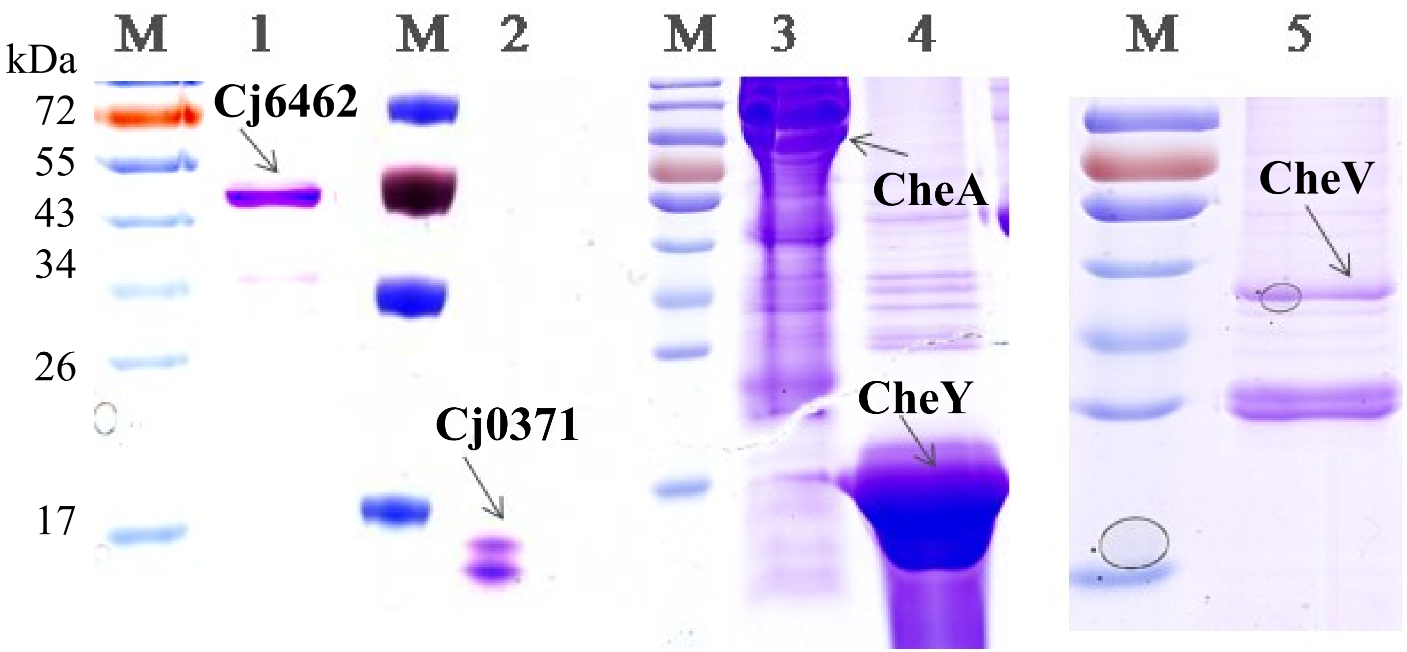

Fig S2 SDS-PAGE of the purified recombinant protein
M. pre-stain marker
1. Purified recombinant protein of rHis-Cj6462；
2. Purified recombinant protein of rHis-Cj0371；
3. Purified recombinant protein of rHis-CheA；
4. Purified recombinant protein of rHis-CheY;
5. Purified recombinant protein of rHis-CheV.
