# Supplementary material for: The Novel Protein Cj0371 Inhibits Chemotaxis of Campylobacter jejuni
Source: Front Microbiol. 2018 Aug 15;9:1904. doi: 10.3389/fmicb.2018.01904 (PMC6104132; doi:10.3389/fmicb.2018.01904)
Supplement: Supplementary file 6 [file Image_3.PDF]

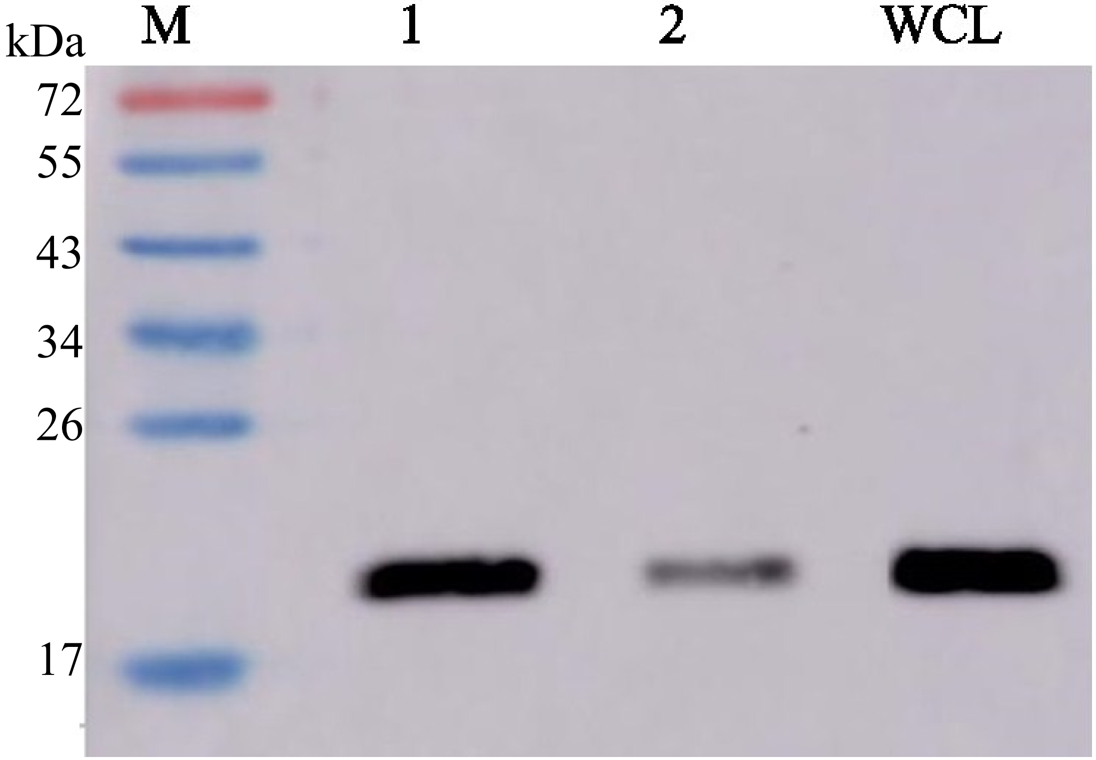

Fig S3 Subcellular localization of the Cj0371 protein by western blot

M. Pre-stain Marker;

1. Cj0371 in the cytoplasm of *C. jejuni*;

2. Cj0371 in the cell membrane of *C. jejuni*;

WCL. The whole cell lysate of *C. jejuni*.
